# Neuron-Oligodendrocyte Interactions in the Structure and Integrity of Axons

**DOI:** 10.3389/fcell.2021.653101

**Published:** 2021-03-08

**Authors:** Greg J. Duncan, Tyrell J. Simkins, Ben Emery

**Affiliations:** ^1^Jungers Center for Neurosciences Research, Department of Neurology, Oregon Health & Science University, Portland, OR, United States; ^2^Vollum Institute, Oregon Health & Science University, Portland, OR, United States; ^3^Department of Neurology, VA Portland Health Care System, Portland, OR, United States

**Keywords:** oligodendrocyte, remyelination, axonal degeneration, demyelination, multiple sclerosis, Wallerian degeneration, mitochondria

## Abstract

The myelination of axons by oligodendrocytes is a highly complex cell-to-cell interaction. Oligodendrocytes and axons have a reciprocal signaling relationship in which oligodendrocytes receive cues from axons that direct their myelination, and oligodendrocytes subsequently shape axonal structure and conduction. Oligodendrocytes are necessary for the maturation of excitatory domains on the axon including nodes of Ranvier, help buffer potassium, and support neuronal energy metabolism. Disruption of the oligodendrocyte-axon unit in traumatic injuries, Alzheimer’s disease and demyelinating diseases such as multiple sclerosis results in axonal dysfunction and can culminate in neurodegeneration. In this review, we discuss the mechanisms by which demyelination and loss of oligodendrocytes compromise axons. We highlight the intra-axonal cascades initiated by demyelination that can result in irreversible axonal damage. Both the restoration of oligodendrocyte myelination or neuroprotective therapies targeting these intra-axonal cascades are likely to have therapeutic potential in disorders in which oligodendrocyte support of axons is disrupted.

## Introduction

Structural variants of the myelin sheath have arisen several times during evolution as a means to allow for the rapid conduction of nerve impulses along axons, including in vertebrates and some species of worm and shrimp (Roots, 2008). Within the central nervous system (CNS) of jawed vertebrates, myelination is carried out by oligodendrocytes, a highly specialized glial cell (Zalc, 2016). The promotion of the speed and efficiency of action potentials has been the best understood purpose of oligodendrocytes and myelin over the past seven decades (Hartline and Colman, 2007). While this role is undeniably important, there is also an increasing appreciation that neurons require support by glia, including oligodendrocytes, for their long-term integrity. When oligodendroglial support is lost, axons become progressively compromised and vulnerable to loss. Consequently, remyelination strategies are being pursued in diseases such as multiple sclerosis (MS) with the hope of not only recovering nerve conduction in the short term but also protecting axons against degeneration in the long term (Franklin et al., 2012; Franklin and Ffrench-Constant, 2017; Lubetzki et al., 2020b). In this review, we outline evidence emerging from both animal models and human pathology that suggests that the integrity of myelinated neurons is dependent on oligodendrocyte support. We focus on recent advances in our understanding of the cellular mechanisms by which oligodendrocytes support axonal and neuronal integrity, how neurons adapt to demyelination, and the intra-axonal cascades contributing to their degeneration.

## Demyelination and Neuronal Damage; Beyond Classical Demyelinating Diseases

Disruption of oligodendrocyte-axon contact and demyelination causes dysfunction in a wide-range of neurological pathologies. MS is considered the prototypical CNS demyelinating disease and features myelin loss in lesions throughout the gray and white matter. Clinically, MS typically presents as a relapsing-remitting (RRMS) course of neurologic dysfunction, though occasionally people with MS experience a progressive accumulation of neurologic disability with few or no relapses (primary progressive MS, PPMS). Demyelination is not the only sequalae in MS; there is also considerable brain atrophy (De Stefano et al., 2010), which is reflective of ongoing axonal damage and neurodegeneration. Acute demyelinating lesions have the highest rate of axonal damage and the degree of damage is correlated with inflammation (Ferguson et al., 1997; Bitsch et al., 2000; Kornek et al., 2000; Kuhlmann et al., 2002). Cytotoxic T-cells found within these lesions can directly damage neurons (Medana et al., 2001; Yan et al., 2003; Shriver and Dittel, 2006) and drugs targeting the activation and infiltration of adaptive immune cells in the CNS are effective at reducing relapses (Derfuss et al., 2020). However, these drugs typically fail to prevent the accumulation of progressive disability during chronic phases of the disease (secondary progressive, SPMS), when acute T-cell mediated lesions wane. Instead, it is the widespread loss of axons and neurons during the progressive phase of the disease that drives permanent disability (Fu et al., 1998; Bjartmar et al., 2000; De Stefano et al., 2001). Crucially, remyelination fails to regenerate myelin along the majority of demyelinated axons leaving them chronically demyelinated (Patrikios et al., 2006; Goldschmidt et al., 2009). These chronically demyelinated inactive lesions constitute the bulk of lesions in MS (Frischer et al., 2015) and display signs of ongoing axonal damage (Kornek et al., 2000). Axonal damage in these chronically demyelinated lesions is, therefore, likely the central contributor to persistent disability in MS.

The susceptibility of axons to damage following myelin and oligodendrocyte loss is observed in other demyelinating pathologies as well. Leukodystrophies are a heterogeneous group of genetic disorders characterized by abnormalities in the development or maintenance of CNS myelin (Kohler et al., 2018; van der Knaap et al., 2019; Wolf et al., 2020). Adrenoleukodystrophy (ALD) is an X-linked demyelinating leukodystrophy caused by mutation in the *ABCD1* gene (Mosser et al., 1993), which encodes an ATP-binding cassette transporter necessary for very long-chain fatty acid (VLCFA) transport into the peroxisome for degradation (McGuinness et al., 2003; van Roermund et al., 2011; Wiesinger et al., 2013). As a consequence, there is an accumulation of VLCFA in cells leading to increased oxidative stress culminating in myelin loss as well as progressive axonal degeneration (Powers et al., 2000; Fourcade et al., 2008). Whether the progressive axonal degeneration occurring in ALD is a primary or secondary to myelin degeneration has not been fully determined. However, peroxisome impairment via the deletion of *Pex5* selectively from oligodendrocyte lineage cells results in the accumulation of VLCFA and axonal degeneration, demonstrating that axonal degeneration can, in principal, be secondary to oligodendrocytic peroxisomal dysfunction (Kassmann et al., 2007). Pelizaeus-Merzbacher disease (PMD) is an X-linked hypomyelinating leukodystrophy caused by mutation, deletion or duplication of the *PLP1* gene. *PLP1*, which encodes proteolipid protein (PLP) and its alternative splicing variant DM20, are the most abundant myelin proteins (Jahn et al., 2020). PMD patients typically have a global developmental delay (motor and cognitive) as well as hypotonia, spasticity, and ataxia (Inoue, 2019). *PLP1* gene duplication is the most common cause of PMD (Mimault et al., 1999). Rodent models of homozygous *Plp1* gene duplication result in premature arrest of myelination and oligodendrocyte apoptosis likely as a result of PLP and cholesterol accumulation leading to endoplasmic reticulum (ER) stress (Kagawa et al., 1994; Readhead et al., 1994; Simons et al., 2002; Karim et al., 2007; Elitt et al., 2018). Mice hemizygous for the PLP gene duplications myelinate normally before developing significant demyelination, inflammation and axonal degeneration (Anderson et al., 1998; Ip et al., 2006). Ultrastructural examination of clinical gene duplications also reveal considerable axonal damage and degeneration (Laukka et al., 2016). The examples of PMD and ALD highlight that neuronal integrity is often impaired in genetic demyelinating pathologies.

Myelin loss and axonal damage are also features of a number of neurologic conditions less commonly thought of as myelin-related disorders. In Alzheimer’s disease (AD), white matter damage is one of the earliest pre-clincal pathologic changes (Hoy et al., 2017; Nasrabady et al., 2018). Single-cell and spatial transcriptomic analyses reveal upregulation of genes involved in remyelination in AD brains (Grubman et al., 2019; Mathys et al., 2019; Agarwal et al., 2020; Chen et al., 2020). However, late term senescent plaques may be inhibitory for remyelination and potentially co-op oligodendrocyte progenitor cells (OPCs) into a pro-inflammatory role (Zhang et al., 2019). These human and rodent studies highlight an underappreciated involvement of oligodendrocyte lineage cells in the pathophysiology of AD. Likewise, the neurodegenerative disorder amyotrophic lateral sclerosis (ALS) also features myelin damage. Decreased expression of myelin basic protein (MBP) in the motor cortex and spinal cord is observed in ALS (Kang et al., 2013). Animal models with mutant *Sod1*, which recapitulate the motor neuron loss observed in ALS, have increased oligodendrocyte loss coupled with a failure of new oligodendrocytes to mature (Kang et al., 2013; Philips et al., 2013). In zebrafish models this precedes motor neuron degeneration, suggesting myelin and oligodendrocyte dysfunction is an early pathology in ALS (Kim et al., 2019). Knockout of mutant *Sod1* from OPCs delays motor decline and increases survival time, possibly by restoring oligodendrocyte support to the neuron (Lee et al., 2012; Kang et al., 2013). Beyond ALS and AD, traumatic injuries to both the brain and spinal cord exhibit conduction deficits and acute demyelination (Kakulas, 2004; Guest et al., 2005; James et al., 2011; Marion et al., 2018) which may also strip axons of oligodendrocyte support and leave them vulnerable to degeneration. However, few axons following spinal cord injury remain chronically demyelinated (Kakulas, 2004; Lasiene et al., 2008). Whether this is due to subsequent degeneration of demyelinated axons or efficient remyelination remains unclear (Duncan et al., 2020). Demyelination and oligodendrocyte loss is also observed in ischemic stroke (Rosenzweig and Carmichael, 2013; Sozmen et al., 2016), and improved myelin regeneration is associated with enhanced functional recovery (Sozmen et al., 2016). This suggests a functional role of demyelination in the deficit following ischemia, at least in rodent models. The breakdown of oligodendroglial support to neurons and demyelination may be common to a wide range of disorders. For these reasons, it is crucial to understand how oligodendrocytes shape axonal function and support their long-term survival.

## Myelinating Glia Organize the Axon

Oligodendrocyte progenitor cells differentiate to form mature oligodendrocytes which extend multiple processes that ensheath nearby axons with concentric layers of membrane (for comprehensive reviews on the development and structure of CNS myelin see Aggarwal et al., 2011; Rasband and Peles, 2015; Simons and Nave, 2016; Stassart et al., 2018; Stadelmann et al., 2019). Depending on the CNS region, each oligodendrocyte will myelinate somewhere between 20 and 60 axons, with myelin internodes being from 20 to 200 μm in length (Chong et al., 2012). During myelination, the leading edge of the developing myelin sheath circles repetitively around the axon, remaining closely associated with the axon to pass under previous myelin wraps with each revolution. Meanwhile, the outer wraps extend laterally, with the terminal edges attaching to the axon in a series of loops that ultimately form the paranode. Over time the cytoplasm is excluded from most regions of the myelin, producing compact myelin (Snaidero et al., 2014). Some areas of non-compact myelin remain; the paranodal loops and the innermost “tongue” of myelin adjacent to the axon remain uncompacted, providing an area of oligodendrocyte cytoplasm closely opposed to that of the axon. In addition, cytoplasmic channels extend through the myelin sheath and provide a connection between the oligodendrocyte cell body and the inner myelin layer (Velumian et al., 2011; Snaidero et al., 2014). Although most prominent during development, these cytoplasmic channels remain in the adult (Snaidero et al., 2017) and likely act as an important conduit for organelles and molecules to support the myelin sheath.

**FIGURE 1:**
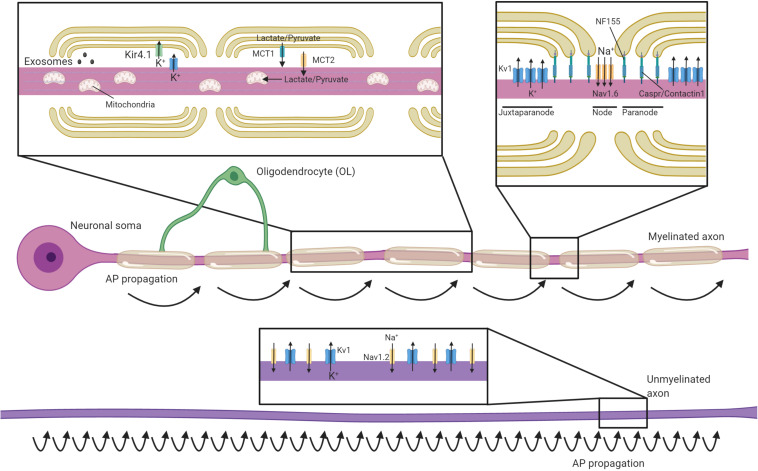
Oligodendrocytes regulate axonal structure, conduction and support their survival. Schematic of an axon myelinated by an oligodendrocyte and an unmyelinated axon. Myelin permits saltatory conductance where action potentials are generated at the nodes of Ranvier. The high membrane resistance and low capacitance generated by the myelin sheath promotes rapid current flow along the myelinated section of the axon to the next node, greatly increasing conduction velocities relative to unmyelinated axons. Oligodendrocytes contact axons at the paranode (via NF155-Caspr/Contactin1) and are crucial for the organization, clustering and maintenance of sodium channels (primarily Nav1.6) at the nodes, as well as Kv1 potassium channels at the juxtaparanodes. In contrast, unmyelinated axons have potassium and sodium (primarily Nav1.2 channels) channels dispersed along the axon and are not confined to discrete excitatory clusters. Oligodendrocytes secrete exosomes that can support neuronal health and buffer potassium via the expression of Kir4.1. Oligodendrocytes provide glycolysis byproducts via monocarboxylate transporters (MCTs), which enter the Krebs cycle and are critical to support axonal metabolism.

An important role of myelin is to establish distinct axonal domains (recently reviewed in detail in Lubetzki et al., 2020a; Rasband and Peles, 2021). The myelinated regions of axons can be divided into subdomains; the internode (corresponding to the compacted region of myelin), the paranodes (where the outer loops of the myelin contact the axon), the node of Ranvier (the ∼1 μm gap between adjacent myelin internodes) and the juxtaparanode (the interface between the paranode and compact myelin, rich in potassium channels) (Figure 1). During development, the clustering of voltage-gated sodium and potassium channels to these domains coincides with the process of myelination, and is disrupted in dysmyelinating mutants (Rasband et al., 1999; Mathis et al., 2001; Arroyo et al., 2002). Several partially redundant mechanisms act in parallel to drive the clustering of nodal proteins during myelination (Susuki et al., 2013). Firstly, glial secreted proteins such as Contactin 1, Phosphocan, and Tenascin-R help cluster axonal neurofascin 186 and voltage-gated sodium channel subunits to sites of future nodes (Kaplan et al., 1997; Susuki et al., 2013; Freeman et al., 2015; Dubessy et al., 2019; Thetiot et al., 2020). Subsequently, oligodendroglial neurofascin 155 interacts with axonal Contactin 1 and Caspr at the paranodes, establishing septate-like junctions that form a barrier between the axon and the myelin loops that prevents the lateral diffusion of nodal proteins (Rasband et al., 1999; Charles et al., 2002; Susuki et al., 2013). Finally, the nodal and paranodal complexes are stabilized through interactions with the axonal cytoskeleton. These interactions are dependent on proteins such as AnkyrinG and protein 4.1B, which tether nodal and paranodal proteins to axonal spectrins, respectively (Komada and Soriano, 2002; Susuki et al., 2013; Brivio et al., 2017). Voltage-gated potassium channels are predominantly restricted to the juxtaparanodal regions through interactions between glial Contactin 2 and axonal Caspr2 (Poliak et al., 2003; Traka et al., 2003). This localization of voltage-gated ion channels essentially restricts the regeneration of action potentials to the node of Ranvier, with current then flowing longitudinally along the myelinated segments of the axon (Hartline and Colman, 2007; Figure 1). This process, termed saltatory conduction, dramatically increases conduction velocity relative to axonal size (Waxman and Bennett, 1972). Although most work has focused the longitudinal current flow along the interior of the axon, a recent study indicates that conduction velocity is also influenced by current flow through the periaxonal space between the innermost myelin wrap and the axon (Cohen et al., 2020). This may prove to be highly significant in light of findings that the width of this space is regulated by neuronal activity and during learning (Cullen et al., 2021).

## Oligodendrocyte Support of Axonal Health

### Oligodendrocytic Shuttling of Monocarboxylates and Glucose to the Axon

Although the best understood roles of myelin are to allow for rapid saltatory conduction by electrically insulating the axon and establishing distinct axonal domains, myelination also has profound implications for axonal energy demand and metabolism. Myelination reduces axonal capacitance and substantially decreases the amount of energy required to restore the resting ion concentrations after each depolarization (Harris and Attwell, 2012). However, myelin, with its concentric membranes, also requires considerable resources to produce and maintain. The act of myelination can be therefore be seen as shifting some of the metabolic load of neuronal firing from axons onto oligodendrocytes. Even though it has been estimated that it may take 15–23 million action potentials for the relative decrease in energy expenditure by a myelinated axon to offset the energetic cost of making the myelin sheath (Harris and Attwell, 2012), this investment may become beneficial at times of high activity where an unmyelinated axon may otherwise struggle to support successive rounds of repolarization through the activity of the Na+/K+-ATPase. Nevertheless, as noted in several reviews on the subject (Mierzwa et al., 2010; Nave, 2010; Stassart et al., 2018), the presence of myelin comes with the liability of impeding the axon’s ability to take up glucose and other metabolites directly from the extracellular space, as myelin restricts diffusion of most molecules to gaps at the paranodal junctions. Combined with the substantial logistical difficulty of transporting energy sources from the neuronal cell body, myelinated axons may rely on metabolic support from the myelinating glia to meet their energy requirements. Oligodendrocytes express the monocarboxylate transporter MCT-1 and can transfer glycolysis products such as lactate to axons (Figure 1), where it can be converted to ATP (Funfschilling et al., 2012; Lee et al., 2012). MCT-1 is expressed both within the myelin sheath and along the adaxonal surface, ideally placing it to directly supply myelinated axons with energy sources they would otherwise be isolated from Rinholm et al. (2011); Lee et al. (2012). Within the corpus callosum, oligodendrocytes support axonal metabolic function by providing glucose as well as glycolysis products (Meyer et al., 2018). Whether the relative oligodendroglial supply of glucose verses the supply of lactate to axons varies across different regions of the CNS is yet to be systematically investigated. Intriguingly, oligodendrocyte expression of the GLUT1 glucose transporter is regulated by NMDA receptor activity (Saab et al., 2016). Given oligodendrocytes do not store glycogen, this suggests a mechanism by which this oligodendroglial uptake of glucose and subsequent supply of glycolysis products might be matched to levels of activity in the myelinated axons (Micu et al., 2016), supporting axons during times of heightened metabolic load (Micu et al., 2017). This feature of supporting axons through provision of glycolysis products would mirror the role of astrocytes and invertebrate glial cells (Pellerin and Magistretti, 1994; Tekkok et al., 2005; Volkenhoff et al., 2015).

Global heterozygosity of the *Slc16a1* gene (which encodes the MCT-1 transporter) in mice causes axonal pathology by eight months of age, including axonal swellings, degeneration, and enlarged axonal mitochondria (Lee et al., 2012). In contrast, a more recent study found that conditionally ablating *Slc16a1* within mature oligodendrocytes (using MOG-Cre) resulted in a more modest and delayed axonopathy, becoming apparent from postnatal day 750 (Philips et al., 2021). This suggests that some of the neurodegeneration in the global heterozygous mice is likely secondary to expression of monocarboxylate transporters in cell types other than myelinating oligodendrocytes, such as astrocytes. Nevertheless, the late-onset axonal pathology seen in oligodendrocyte conditional knockouts of *Slc16a1* clearly indicates that oligodendrocyte provision of metabolites is required for axonal integrity, at least in the aging CNS (Philips et al., 2021). Given (Lee et al., 2012) found reduced expression of MCT1 in the cortex of ALS patients and oligodendrocytes of SOD1 mutants it is tempting to speculate that enhancing oligodendrocyte provision of glycolysis products to axons could be neuroprotective in disease contexts. A recent study seeking to virally overexpress MCT1 in oligodendrocytes in the model failed to find any therapeutic benefit, however (Eykens et al., 2021). This highlights the need for a better understanding of the situations in which oligodendrocyte provision of glycolysis products is a limiting factor for axonal survival.

### Extracellular Vesicle Transfer to Axons

Extracellular vesicles (EVs) are lipid bilayer-bound structures that can carry a variety of cargos including metabolites, proteins, lipids, mRNAs, and miRNAs. Based on size and release mechanisms, they are classed as exosomes [30–100 nm, released from multivesicular bodies (MVBs)] or microvesicles (100–1,000 nm, released by budding of the plasma membrane). Once secreted, EVs can be taken up by other cells, where they modulate cellular phenotypes and gene expression (Holm et al., 2018). Increasing evidence indicates that oligodendrocyte-derived EVs support neuronal integrity. Oligodendrocyte MVBs, the precursor to released exosomes, are concentrated in regions of non-compact myelin and at the adaxonal loop (Hsu et al., 2010; Fruhbeis et al., 2013). Following secretion, oligodendrocyte EV’s are taken up by neurons (Kramer-Albers et al., 2007; Fruhbeis et al., 2013; Mukherjee et al., 2020), and promote their survival, at least in culture (Fruhbeis et al., 2013). Conversely, mice with conditional oligodendrocyte ablation of Rab35 (required for secretion of exosomes from oligodendrocyte MVBs) display progressive oxidative damage and neuronal loss (Hsu et al., 2010; Mukherjee et al., 2020). Somewhat like the expression of glucose transporters (see section “Oligodendrocytic Shuttling of Monocarboxylates and Glucose to the Axon”), the release of EVs from oligodendrocytes is stimulated by activation of oligodendroglial glutamate receptors (Fruhbeis et al., 2013). The release of EVs along the periaxonal space would presumably allow for preferential uptake of oligodendrocyte-released EVs by the myelinated axon, allowing for a relatively targeted and activity-regulated transfer of supportive metabolites or signaling molecules. Such a transfer has been demonstrated to occur between ensheathing glia and the giant squid axon (Buchheit and Tytell, 1992), and likely represents an evolutionarily conserved relationship between glia and axons.

A large number of details remain to be determined about the role of oligodendrocyte exosomes, including their cargo. Oligodendrocyte-derived exosomes are known to be enriched in chaperone proteins and enzymes mediating protection against oxidative stress (Kramer-Albers et al., 2007), but thus far individual components has received little experimental attention. One notable exception is ferritin heavy chain, which is secreted with oligodendrocytes EVs and protects neurons against ferroptotic cell death *in vitro* (Mukherjee et al., 2020). Oligodendrocyte conditional ablation of the *Fth1* gene in mice resulted in neuronal loss and oxidative damage, indicating that at least some of the neurodegeneration seen in the oligodendrocyte Rab35 conditional knockouts may be secondary to loss of ferritin heavy chain secretion (Mukherjee et al., 2020). It should be noted that oligodendroglial expression of ferritin heavy chain is also required for early postnatal myelination (Wan et al., 2020), which raises the potential confounder that some of the axonal degeneration in the *Fth1* condition knockout mice could be secondary to myelin defects. Nevertheless, with conditional ablation of the *Fth1* gene in adulthood the neuronal loss occurred in the absence of detectable disruption to myelin (Mukherjee et al., 2020), arguing against this interpretation. Reduced EV release is seen in oligodendrocytes derived from *Plp1* and *Cnp1* null mice, both of which display progressive axonal degeneration (Frühbeis et al., 2020). Together, these results identify the secretion of EVs from oligodendrocytes as a potentially important mechanism for axonal support by oligodendrocytes.

### Potassium Buffering and the Glial Syncytium

Action potentials rely on differential concentrations of Na^+^ and K^+^ ions across the neuronal membrane, with the extracellular space being relatively high in Na^+^ and low in K^+^. The repolarization phase of each action potential releases K^+^ into to the extracellular space, which needs to be removed by a network of glial cells to enable subsequent action potentials (Rash, 2010). Astrocytes fulfill this function throughout much of the CNS. However in myelinated fibers, voltage-gated K^+^ channels are primarily localized to the juxtaparanodal region where they can release K^+^ into the periaxonal space underlying the myelin (Wang et al., 1993; Poliak et al., 2003). Oligodendrocytes express the inward-rectifying potassium channel Kir4.1 (coded by the *Kcnj10* gene) at the perinodal area and along the inner myelin tongue, where it would be well placed to clear K^+^ from the periaxonal space (Schirmer et al., 2018; Figure 1). Indeed, oligodendrocyte *Kcnj10* conditional knockouts display delayed K^+^ clearance from the white matter, deficits in high-frequency axonal firing, and seizures (Larson et al., 2018; Schirmer et al., 2018). Interestingly, the failure of oligodendrocytes to clear potassium from the periaxonal space is also crucial for the long-term health of axons. *Kcnj10* conditional knockouts display axonal mitochondrial swelling and degeneration in long white matter tracts such as the spinal cord and optic nerve and loss of retinal ganglion cells (RGCs; Schirmer et al., 2018). Oligodendrocytes may rely heavily on gap junctions to siphon potassium away from the inner myelin tongue and into the broader glial syncytium. Heterotypic gap junctions form between oligodendrocytes and astrocytes, predominantly through either oligodendrocyte Cx47 and astrocyte Cx43 or oligodendrocyte Cx32 and astrocyte Cx30 (Kamasawa et al., 2005; Orthmann-Murphy et al., 2007; Magnotti et al., 2011). These heterotypic junctions could mediate the directional shuttling of potassium from the oligodendrocytes into the astrocytes (Fasciani et al., 2018), though oligodendrocyte-oligodendrocyte gap junctions may also help disperse potassium (Kettenmann et al., 1983; Battefeld et al., 2016).

It is likely that the roles of connexin-based channels between glia could extend well beyond potassium buffering. For example, *in vitro* studies demonstrate that labeled glucose analogs can be trafficked between oligodendrocytes and astrocytes, raising the possibility that gap junctions could mediate similar trafficking of glucose between astrocytes and oligodendrocytes *in vivo* (Niu et al., 2016). Consistent with this idea, genetic disruption of Cx47, necessary to fully connect oligodendrocytes to astrocytes, blocks the ability of glucose-loaded corpus callosum oligodendrocytes to support axonal firing in conditions of oxygen and glucose deprivation (Meyer et al., 2018). Similarly, loading thalamic astrocytes with glucose or lactate supports postsynaptic activity during oxygen and glucose deprivation, a protective effect that is blocked by disruption of Cx32 and Cx47 (Philippot et al., 2021). Together, these data indicate gap junctions may serve to link glial networks and distribute metabolites that are ultimately shuttled to axons through oligodendrocytes. Recent findings indicate that astrocytes and oligodendrocytes may also act in concert to regulate the breakdown of glutamate and redistribution of its metabolites, with subsets of oligodendrocytes in the spinal cord and midbrain expressing glutamine synthetase (Philippot et al., 2021). Nevertheless, the metabolites that are trafficked between astrocytes and oligodendrocytes through gap junctions in more physiological contexts have been challenging to experimentally determine, especially given the profound myelin deficits seen in mice lacking Cx47 and Cx32 (Menichella et al., 2003). The exact role of these glial gap junctions in supporting axonal health is likely to be an ongoing area of important work.

## Relationship Between De/Dysmyelination and Axonal Loss in Rodent Models

Demyelinating and dysmyelinating animal models have offered key insights into how oligodendrocytes support neurons. Demyelination in rodents is typically produced in one of three ways; via autoimmune attack against myelin, administration of demyelinating toxins, or genetic depletion of oligodendrocytes. These strategies differ dramatically in the extent of myelin and axonal damage as well as the aspects of MS modeled. A key shared characteristic of these models is the fairly rapid and effective remyelination. At this time no model accurately mimics the stresses placed on the axon by long-term remyelination failure like that seen in MS. Nevertheless, these models have revealed much about the extent to which oligodendrocytes contribute to neuronal integrity.

### EAE

Experimental autoimmune encephalitis (EAE) is a family of models in which the immune system is activated to target the myelin sheath for degradation. Typically this is achieved by the transfer of myelin-reactive T cells or the administration of myelin peptides alongside adjuvants to drive the immune response (Kipp et al., 2012; Ransohoff, 2012; Lassmann and Bradl, 2017). In recent years, the most common model of EAE involves immunization with the 35–55 peptide of myelin oligodendrocyte glycoprotein (MOG_35__–__55_), a myelin protein found in the outermost lamellae (Mendel et al., 1995), along with complete Freund’s adjuvant and pertussis toxin. In C57BL/6 mice this reliably causes inflammatory demyelinating lesions, mostly within the spinal cord, which are characterized by CD4+T-cell infiltration (Soulika et al., 2009; Berard et al., 2010). Significant axonal damage and subsequent transection occur within the spinal cord (Kim et al., 2006; Aharoni et al., 2011), and are correlated with persistent decline (Wujek et al., 2002; Papadopoulos et al., 2006). Both transport deficits and swellings are observed prior to demyelination and the extent of axonal damage is closely correlated with, and driven by inflammatory infiltrate (Soulika et al., 2009; Nikic et al., 2011; Sorbara et al., 2014). Nevertheless, genetic manipulations of the oligodendrocyte lineage to improve remyelination also enhance axonal preservation following EAE (Mei et al., 2016), indicating oligodendroglial and myelin damage likely contributes to axon loss in this model. However, the stochastic nature of demyelinating lesions in EAE and challenges in uncoupling immunomodulatory effects from remyelination make it difficult to use this model to make mechanistic insights about how oligodendrocytes support axons.

### Toxic Models

Toxin-induced models of demyelination, such as dietary cuprizone or focal injections of lysolecithin (or less commonly ethidium bromide) offer strict spatial and temporal control over demyelination and subsequent remyelination. Lysolecithin is typically injected into white matter tracts where it acts as a detergent to disrupt membranes and induce demyelination (Plemel et al., 2018). This is followed by rapid remyelination of remaining axons (Woodruff and Franklin, 1999). While this model has been an enormously important tool to understand the mechanisms of remyelination, lysolecithin does not act selectively on myelin membranes, but disrupts all membranes often killing astrocytes and triggering calcium accumulation and subsequent degeneration of axons (Zhao et al., 2015; Plemel et al., 2018). The combination of very rapid remyelination, small lesion size and axon damage through the direct action of the toxin makes lysolecithin-mediated demyelination an unsuitable model for delving in to how oligodendrocytes support neuronal integrity. Conversely, cuprizone can cause prolonged demyelination throughout the corpus callosum, hippocampus, cortex and the cerebellum for as long as the animals are fed cuprizone (Matsushima and Morell, 2001; Gudi et al., 2014; Bai et al., 2016; Zhan et al., 2020). Cuprizone is a copper chelator, but the precise mechanism by which cuprizone induces demyelination is unclear, though oxidative damage to oligodendrocyte is seen within days of administration of cuprizone followed by both apoptotic and non-apoptotic forms of cell death (Buschmann et al., 2012; Skripuletz et al., 2013; Jhelum et al., 2020). At higher doses, cuprizone is widely toxic to cells, inducing hepatoxicity and spongiform encephalopathy (Suzuki, 1969; Suzuki and Kikkawa, 1969), though damage is most prominent in oligodendrocytes at the doses typically given to induce demyelination. Axon degeneration can be severe with between 20% and 50% of the axons lost in the corpus callosum depending on the length and dose of cuprizone administered (Irvine and Blakemore, 2006; Manrique-Hoyos et al., 2012). Interestingly, following withdrawal of cuprizone from the diet there is ongoing damage to axons despite remyelination (Lindner et al., 2009) ultimately culminating in reduced axon number and motor coordination (Manrique-Hoyos et al., 2012). This indicates that even with successful remyelination ongoing axonal injury can occur. Whether the failure to fully protect axons after remyelination is due to an inherent inability of new, remyelinating oligodendrocytes to adequately support axons, an ongoing cytotoxic inflammatory state induced by cuprizone, or persistent toxicity remains unclear.

### Genetic Models

Demyelination can be induced by the genetic targeting of the oligodendrocyte lineage, which avoids the direct action of inflammation or toxins on neurons. This is typically achieved via the inducible expression of “suicide” genes in oligodendrocytes such as diphtheria toxin subunit A (DTA; Traka et al., 2010; Pohl et al., 2011), DTA receptor in conjunction with diphtheria toxin (Oluich et al., 2012), activated caspases (Caprariello et al., 2012), or via the deletion of a key gene for myelin or oligodendrocyte maintenance like *Myrf* (Koenning et al., 2012; Hartley et al., 2019). Axon swellings are observed prior to and during outright demyelination in the DTA models (Pohl et al., 2011; Oluich et al., 2012) but axonal loss is not observed, at least within the visual system (Traka et al., 2010). Following demyelination, the number of oligodendrocytes recovers and the mice rapidly remyelinate in DTA models (Traka et al., 2010; Pohl et al., 2011). Similarly, genetic mutants that congenitally lack compact myelin such as the *Mbp* mutant *shiverer* mice or *les* rats, do not have progressive axonal loss, and retain ensheathing oligodendrocytes (Rosenbluth, 1980; Griffiths et al., 1998; Smith et al., 2013). These studies illustrate a critical point – loss of myelin *per se* does not invariably lead to the degeneration of the underlying axon when oligodendroglial support is restored rapidly or retained (as in the case of *Mbp* mutants). Additionally, the cell-specificity of these genetic models will likely be highly beneficial to elucidate mechanisms by which oligodendrocytes support axons, particularly if models with delayed oligodendrogenesis and remyelination can be developed.

### Long-Term Breakdown of Oligodendrocyte Support in Myelin Gene Knockouts Leaves Neurons Vulnerable to Damage

Substantial demyelination in animal models does not invariably lead to neurodegeneration, conversely, germline knockout of several genes expressed solely within the oligodendrocyte lineage go on to develop axonal degeneration despite forming normal levels of myelin. *Plp1* null mice develop compact myelin but lack stable intermembrane bonding resulting in separation of the myelin lamellae (Boison and Stoffel, 1994; Rosenbluth et al., 1996; Coetzee et al., 1999). However, outright demyelination remains rare even with aging (Klugmann et al., 1997; Luders et al., 2019). These mice develop profound axonal transport defects and progressive axonal loss, particularly in long and thin axons (Griffiths et al., 1998; Garbern et al., 2002; Edgar et al., 2004). Axonal spheroids, indicative of axonal damage, are observed by four months of age in germline knockouts of *Plp* (Griffiths et al., 1998), and four months following tamoxifen administration in inducible oligodendrocyte-specific knockouts (Luders et al., 2019). Axonal spheroids precede T-cell mediated infiltration though are coincident with astrogliosis and microglial activation (Luders et al., 2017, 2019). Similarly, mice lacking the *Cnp1* gene have progressive axonal loss culminating in considerable axonal degeneration in the brain and shortened lifespan (Lappe-Siefke et al., 2003; Edgar et al., 2009). Comparable to the *Plp* null mice, axon loss begins by about four months of age (Lappe-Siefke et al., 2003; Edgar et al., 2009). CNP is expressed in the inner, non-compact tongue of myelin (Braun et al., 1988; Trapp et al., 1988). Its knockout swells the inner tongue, but compact myelin thickness is normal and in early adulthood (P60) has equivalent numbers of myelinated axons (Edgar et al., 2009). So, what explains this apparent discrepancy – substantial demyelination and oligodendrocyte apoptosis throughout the CNS does not necessarily lead to axon degeneration, yet a number of single gene knockouts in oligodendrocytes do? In the case of toxin and genetic models of demyelination oligodendrocyte support is usually rapidly restored through oligodendrogenesis and remyelination with the bulk of remyelination occurring within two weeks in focal chemical models of demyelination (Duncan G. J. et al., 2017) and within two months in cuprizone demyelination (Sachs et al., 2014) and oligodendrocyte depletion (Traka et al., 2010). In contrast, within the *Plp* knockouts and *Cnp1* null mice oligodendrocyte-axon interactions may be impaired throughout lifespan with axon loss taking at least four months to accrue. In both *Plp* and *Cnp* knockout mice there is considerable evidence oligodendrocyte-axon interactions are impeded. *Plp*-null oligodendrocytes are outcompeted for axons by wildtype oligodendrocytes (Yool et al., 2001). This is especially true for small diameter axons, which are preferentially vulnerable in *Plp* null mice (Yool et al., 2001; Nave and Trapp, 2008). PLP may aide in the extension of processes and ensheathment of axons, which is necessary for the long-term stability of oligodendrocytes following differentiation (Hughes et al., 2018) and likely indicates a diminishment of oligodendrocyte-axon support in *Plp* null mice. Likewise, CNP is known to maintain the opening of cytoplasmic channels in myelin (Snaidero et al., 2017). If these channels are compromised in the *Cnp1* null mice, oligodendrocyte support functions such as delivery of EVs (Fruhbeis et al., 2020) or lactate (Funfschilling et al., 2012; Lee et al., 2012) could be disrupted, leaving the myelinated portions of the axons stressed and vulnerable to loss. These studies indicate that, a long-term breakdown of normal oligodendroglial support, even if the myelin sheath is broadly maintained, can trigger axon loss.

### Does Remyelination Restore Neuronal Health and Function?

With remyelinating therapies entering clinical trials (Green et al., 2017; Plemel et al., 2017; Stangel et al., 2017; Cadavid et al., 2019; Lubetzki et al., 2020b) it will be important to establish whether new oligodendrocytes are capable of supporting axonal integrity and function to a similar degree as those formed during development. Experimentally, there is evidence remyelinating oligodendrocytes may not confer the same level of support to neurons. In the cuprizone model of demyelination, axon loss can continue despite accomplished remyelination and cessation of cuprizone administration (Manrique-Hoyos et al., 2012). Remyelinated axons have higher mitochondrial content, suggesting that metabolic support may not be fully restored and a greater share of the energetic burden remains on the neuron (Zambonin et al., 2011). The myelin of adult-born oligodendrocytes is often thinner and internodes are typically shorter than those derived during development (Lasiene et al., 2009; Young et al., 2013; Duncan I. D. et al., 2017). This may contribute to the persistent motor deficits in genetic models of demyelination despite considerable levels of remyelination (Hartley et al., 2019). One approach to determine how effective remyelination is in protecting axons and restoring function has been to use the relative heterogeneity in remyelination in MS as a natural experiment. The level of axon damage between the subset of lesions capable of remyelination in MS – so called shadow plaques – is less than those that remain chronically demyelinated (Kornek et al., 2000; Kuhlmann et al., 2002). Likewise, to examine remyelination’s role in recovery in MS, a recent study used positron emission tomography with a compound sensitive to myelin changes (Pittsburgh compound B) to characterize the extent of remyelination relative to disability (Bodini et al., 2016). Those with greater levels of remyelination had lower levels of disability (Bodini et al., 2016). It is important to note that these studies, while informative, represent only correlative evidence that remyelination is protective and restores function, not causal data. It remains possible that axons less-damaged during inflammatory demyelination are more receptive to remyelination in MS, which could also explain these associations.

Experimentally, only a few animal studies have attempted to determine if remyelination directly improves axonal health. This is in large part due to difficulty in decoupling inflammatory and degenerative processes from that of remyelination. To assess if a failure to remyelinate increases axonal damage, X-irradiation was applied during cuprizone demyelination to deplete OPCs necessary for remyelination (Blakemore and Patterson, 1978; Irvine and Blakemore, 2007). Increased axonal damage, and fewer axons with highly phosphorylated neurofilaments were observed in the corpus callosum, both of which were rescued by transplantation of OPCs capable of remyelinating (Irvine and Blakemore, 2008). However, irradiation-induced changes in both astrogliosis and inflammation confounded whether remyelination failure is the specific causative agent that increased axonal damage. A more cell-specific gain of function approach was recently undertaken to determine the role of remyelination in axonal integrity by deleting the muscarinic receptor 1 from oligodendrocyte lineage cells (Mei et al., 2016). This resulted in more rapid remyelination and a greater number of neurofilament-positive axons following EAE, providing evidence of a neuroprotective role of remyelination. However, whether a failure to remyelinate, like that seen in MS lesions, triggers worsened axonal loss in the absence of autoimmune T-cell infiltration remains untested. Inducible cell-specific knockouts have been used to block *de novo* oligodendrogenesis in adulthood (McKenzie et al., 2014; Schneider et al., 2016; Xiao et al., 2016; Pan et al., 2020) and during repair (Duncan G. J. et al., 2017; Duncan et al., 2018) and may provide a selective approach to determine to what extent remyelination protects axons.

Remyelinated axons typically have shorter internodes and thinner myelin. This begs the question to what extent is remyelination capable of restoring conduction and behavior? Computer simulations indicate the sudden loss of a single myelinated internode along an axon may be sufficient to temporarily block conduction (Koles and Rasminsky, 1972; Waxman and Brill, 1978). In agreement with these simulations, conduction is highly diminished through focal lesions in the days after an injection of lysolecithin or ethidium bromide (Smith et al., 1979; Black et al., 1991). Endogenous remyelination was shown decades ago to improve conduction and increase the speed of propagation relative to demyelinated axons (Smith et al., 1979), a finding that identified remyelination as a truly regenerative process. Remyelination restores sodium channel clustering to the nodes and Kv1.1 and Kv1.2 channels to the juxtaparanode (Rasband et al., 1998; Coman et al., 2006). Nodal structure is critical for action potential propagation and its restoration likely helps reestablish conduction (Arancibia-Carcamo et al., 2017). Perhaps one of the most compelling instances by which remyelination promotes functional recovery is following high-dose radiation injury to rats. High doses of radiation depletes OPCs, induces oligodendrocyte death and causes demyelination (Kurita et al., 2001). Following brain-wide demyelination induced by radiation, injection of human OPCs resulted in considerable remyelination and fully restored performance on a novel object recognition task or the rotarod depending if OPCs are injected into either the corpus callosum (for novel objection recognition) or cerebellum (for rotarod) (Piao et al., 2015). Remyelination is less critical for recovery in instances when axonal damage is high, or the spread of demyelination is low like traumatic injury to the spinal cord (Duncan et al., 2018). In such cases axons are likely able to restore conduction through small areas of demyelination (up to several millimeters in length) (Felts et al., 1997). Remyelination, therefore, seems more likely to propel recovery when the extent of demyelination is high, axon damage is low and the area of remyelination is large.

It is now clear that remyelination is broadly effective at improving nodal structure, conduction, and function at least relative to demyelinated axons. However, two recent publications (Bacmeister et al., 2020; Orthmann-Murphy et al., 2020) have made an interesting observation; the pattern of remyelination in gray matter diverges significantly from that of the initial myelination. Following cuprizone diet administration, the vast majority of myelinated internodes are lost in the upper cortical layers, where axons usually display an intermittent pattern of myelination (Bacmeister et al., 2020; Orthmann-Murphy et al., 2020). Following cessation of cuprizone administration, many axons were remyelinated at near-identical locations to the original internodes (Orthmann-Murphy et al., 2020). Axons which had a higher initial degree of myelination were more likely to be precisely remyelinated, suggesting the preference for myelinating particular neuronal populations can be maintained (Orthmann-Murphy et al., 2020). However, ∼32% of denuded myelin sheaths were not replaced after 8 weeks, with a large number of new myelin sheaths instead being formed along previously unmyelinated axonal segments (Orthmann-Murphy et al., 2020). Even relatively small changes in *de novo* myelination by new oligodendrocytes have significant impacts on motor learning (McKenzie et al., 2014; Xiao et al., 2016), spatial learning (Steadman et al., 2019), and fear conditioning (Pan et al., 2020). This suggests that the altered pattern of cortical myelination seen following remyelination has the potential to disrupt higher-order brain function and synchrony between neuronal circuits. Interestingly, in the motor cortex forelimb reach training increased the proportion of demyelinated internodes that received myelin, suggesting selective activity paradigms may be necessary to reconstitute myelin patterns fully (Bacmeister et al., 2020). Training and rehabilitation might therefore need to be coupled with remyelinating therapeutics to not only increase the quantity but to target myelin to appropriate axons in demyelinating disease.

## Neuronal Adaptations to Demyelination

### Demyelination-Induces Changes to Axonal Ion Channels

**FIGURE 2:**
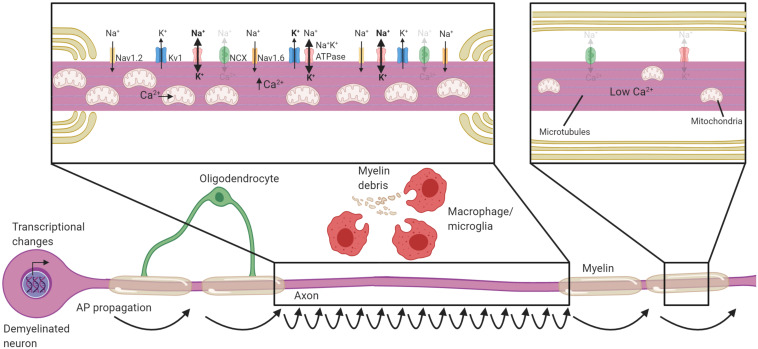
Neuronal adaptations to acute demyelination. Schematic of a partially demyelinated neuron early after demyelination. Conduction is reestablished through the demyelinated segment by the increased expression of sodium channels along the axolemma, but it is notably slower. Demyelinated axons require greater Na^+^ entry to depolarize the axon, necessitating increased activity of the Na^+^K^+^ATPase. Mitochondria increase in number and size within demyelinated axons to meet the higher demand for ATP and also uptake Ca^2+^. If Na^+^K^+^ATPase has sufficient ATP, the NCX is rarely activated in the reverse direction (faded arrows). Transcriptional changes occur within the neuron in response to demyelination and could be critical to these adaptions. Faded text and indicates low activity or levels, **bolded** text or thick arrows indicates increased levels following demyelination.

The disruption of oligodendrocyte-axon interactions during demyelination fundamentally reshapes the organization of excitatory domains along the axon. Breakdown of paranodal contact between the oligodendrocyte and the axon occurs at a very early stage during demyelination in both MS (Wolswijk and Balesar, 2003; Coman et al., 2006; Howell et al., 2006) and in EAE (Fu et al., 2011). The paranode acts as a diffusion barrier necessary to maintain the sodium channels at the node (Rios et al., 2003; Dupree et al., 2004; Zhang et al., 2020), and its loss instigates sodium channels to spread along the axolemma (Rios et al., 2003; Dupree et al., 2004). There is also increased expression of sodium channels (England et al., 1991) including the N_av_1.2 sodium channel following demyelination (Figure 2; Craner et al., 2003, 2004; Coman et al., 2006), which is normally restricted to unmyelinated axons (Caldwell et al., 2000; Boiko et al., 2001; Lubetzki et al., 2020a). The upregulation and increased expression of sodium channels likely has the benefit of restoring conduction through demyelinated segments stretching several millimeters (Felts et al., 1997), and might be crucial to recovery during the relapsing-remitting phase of MS and in rodent models of inflammatory demyelination (Trapp and Nave, 2008). However, demyelinated axons have notably slower conduction, and are more susceptible to generating ectopic action potentials (Smith and McDonald, 1982; Hamada and Kole, 2015). Further, heightened sodium channel expression results in increased axoplasmic Na^+^ accumulation. Sodium must then be removed from the axon for repolarization via the increased operation of Na^+^K^+^ATPase, a highly energetic process (Trapp and Stys, 2009; Harris and Attwell, 2012). Changes to excitatory domains following demyelination may alleviate conduction block, but it culminates in slow, discordant, energy-intensive propagation of APs in the absence of oligodendrocyte myelination.

### Increased Mitochondrial Content in Demyelinated Axons

It is perhaps not surprising that to support the increased energetic burden on the axon after demyelination, mitochondrial content is increased within demyelinated axons (Figure 2). An increase in the number of mitochondria was first detected in experimental demyelinated lesions (Mutsaers and Carroll, 1998; Sathornsumetee et al., 2000), then observed in MS (Sathornsumetee et al., 2000; Zambonin et al., 2011). Increased mitochondrial content and activation of respiratory chains is likely a generic change axons undergo when they lack myelin, as it is also observed in transgenic models like the compact myelin-deficient *shiverer* (Andrews et al., 2006; Joshi et al., 2015). There are both motile and stationary pools of mitochondria in the axon, and stationary mitochondria tend to accumulate in areas of high metabolic demand (Misgeld et al., 2007; Misgeld and Schwarz, 2017; Mandal and Drerup, 2019). A plausible signal for immobilization of mitochondria in demyelinated axons is the activity of Na^+^K^+^ATPase (Zhang et al., 2010; Ohno et al., 2011), linking metabolic requirements to mitochondrial trafficking. Indeed, demyelination increases the number of stationary mitochondria and this likely aids in meeting local metabolic burden of demyelinated axons (Kiryu-Seo et al., 2010). The axonal mitochondrial anchoring protein syntaphilin (Kang et al., 2008) increases in expression following demyelination and is necessary for the immobilization of mitochondria within the axon (Ohno et al., 2014). Following cuprizone demyelination, deletion of syntaphilin results in potentiated axon damage demonstrating the importance of mitochondrial anchoring to areas of high demand (Ohno et al., 2014). Energetic failure within the axon is the likely cause of increased damage in syntaphilin knockouts, as the blockade of sodium channels, and therefore reduced activation of Na^+^K^+^ATPase, ameliorates the axonal damage (Ohno et al., 2014). Neuronal mitochondrogenesis and immobilization of mitochondria to sites of demyelination is therefore necessary to meet the increased energy burden placed on the axon following demyelination.

### Neuronal Transcriptional Responses to Demyelination

Transcriptional changes often underlie differences in cellular function, and gene expression profiling has been undertaken in experimental chemical demyelinating lesions (Lovas et al., 2010) as well as in MS to examine neuronal gene changes (Dutta et al., 2007, 2011; Schirmer et al., 2019). Dutta et al. (2011) took advantage of the variable degree of demyelination within individual MS hippocampi to assess the influence of demyelination on gene expression. There is a significant reduction in the expression of genes regulating axonal transport and synaptic structure in the demyelinated hippocampi relative to myelinated hippocampi in MS and healthy controls (Dutta and Trapp, 2011). Subsequent studies have confirmed that synapse loss is a robust and early event in demyelinating disease (Jurgens et al., 2016; Werneburg et al., 2020) and axon transport is highly impaired by demyelination (Sorbara et al., 2014). There is also a shift toward inhibitory neurotransmission with genes involved in glutamatergic signaling downregulated and increased expression of genes involved in GABAergic neurotransmission following demyelination in the hippocampus (Dutta et al., 2011, 2013). These findings highlight how transcriptional changes can be used to identify physiological changes. One disadvantage of whole-tissue approaches is that they obscure which specific cell types expression changes are found in. Additionally, loss of specific types of cells may bias the differential expression data. Single-cell RNAseq offers an unbiased approach to examine the heterogeneity in gene expression between different cell types or can be used to determine if the cell-type constituents are changing. Single nuclei RNAseq was undertaken on the cortical and adjacent subcorticial white matter lesions from people with MS who did not receive immunomodulatory treatments (Schirmer et al., 2019). There is selective vulnerability of L2/L3 cortical neurons that were *Cux2*+, and these neurons demonstrated enhanced activation of cell-stress pathways and protein folding response (Schirmer et al., 2019). At this point, gene-expression studies comparing demyelinated versus myelinated neurons have uncovered wide-ranging expression changes in axonal transport, synaptic stability, inhibitory neurotransmission and the activation of cell stress pathways, which together reveals that virtually all aspects of their cellular function are altered following demyelination. Future studies should assess whether these transcriptional changes are induced by the inflammatory milieu of MS lesions or are a general consequence of demyelination. In addition, it will be important to functionally determine which of the transcriptional changes in demyelinated neurons are adaptive and which represent maladaptive or pathological changes.

## Mechanisms of Axonal Degeneration Following Demyelination

**FIGURE 3:**
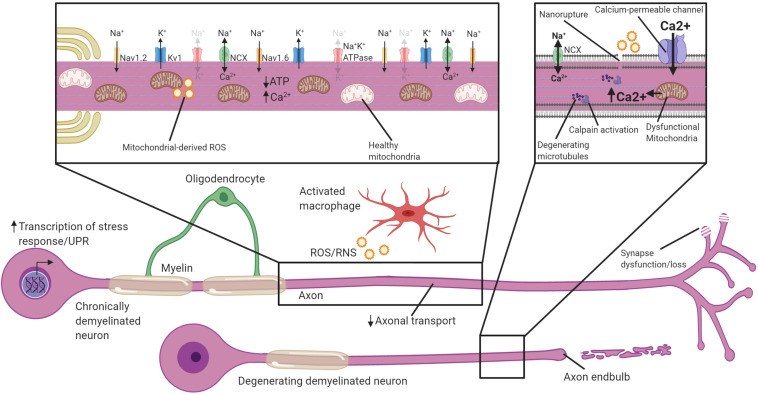
Potential mechanisms by which chronically demyelinated axons degenerate. Schematic of a chronically demyelinated intact axon and an additional demyelinated axon undergoing degeneration. Demyelinated axons are exposed to inflammatory mediators including ROS/RNS in MS, have reduced axonal transport, and may have synapse dysfunction/loss. The chronically demyelinated axon is likely to be in an energy crisis in which the lack of oligodendrocyte support coupled with mitochondrial damage and increased energetic demands to sustain AP propagation means there is a shortfall of ATP necessary to drive the Na^+^K^+^ATPase. This causes a reversal of the NCX to remove Na^+^ from the axon, but at the cost of calcium entry. Disruption of the plasma membrane, and calcium entry through calcium-permeable channels including glutamate receptors, ASICs, VGCC and mitochondrial release cause calcium to accumulate in the axon. Intra-axonal calpains are activated when calcium accumulates to high levels and begin the process of degeneration and breakdown critical cytoskeletal structures including microtubules. Activation of cell-stress pathways and the unfolded protein response also are present in many demyelinated neurons that are susceptible to degeneration. Faded text and indicates low activity or levels, **bolded** text or thick arrows indicates increased activated.

Neurons undergo swift changes in response to demyelination by altering their transcription, distribution of their excitatory domains, and energy metabolism. While these changes may be necessary for restoration of some level of conductance through demyelinated segments, the lack of oligodendrocytic support and increased energetic demands nevertheless leave neurons vulnerable to damage, particularly if not remyelinated over extended periods. Over the next several sections we will discuss potential mechanisms of axonal degeneration following demyelination. We will focus on how ion channel redistribution puts increased metabolic strain on the neuron, and when coupled with inflammatory mediators, oxidative damage, transport deficits and mitochondrial dysfunction, the axon is left with an energy deficit and ultimately is vulnerable to degeneration (Figure 3). We will also discuss the evidence that active “death signaling” pathways identified from studies of Wallerian degeneration may be involved in demyelination-associated axonal degeneration.

### Axoplasmic Calcium Overload in Demyelinated Axons Triggers Axonal Degeneration

Action potential propagation in axons without myelin requires greater influx of Na^+^ to overcome the higher capacitance and subsequently change potential. To repolarize the demyelinated axon, increased activity of the Na^+^K^+^ ATPase is needed to extrude the Na^+^ (Trapp and Stys, 2009). The increased flux of Na^+^ into demyelinated axons coincident with inflammation in MS has been hypothesized to cause an energy run-down within the axon, reminiscent of hypoxia (Trapp and Stys, 2009; Lassmann et al., 2012; Friese et al., 2014). In anoxic conditions, which greatly limit ATP production, Na^+^ accumulation in the axon drives a reversal of the sodium-calcium exchanger (NCX) and a buildup of intracellular calcium ions (Ca^2+^) (Stys et al., 1991, 1992). Calcium overload has long been identified as a general initiator of axonal degeneration, as calcium ionophores can directly drive axonal degeneration, while calcium chelators delay axonal degeneration following transection *in vitro* (Schlaepfer and Bunge, 1973; George et al., 1995). In EAE, chelation of extracellular calcium almost completely ameliorates axonal degeneration, at least over the short-term (Witte et al., 2019). The accumulation of calcium propels axonal degeneration by activating intra-axonal proteases like calpains, which break down cytoskeletal elements such as neurofilaments and microtubules (Kamakura et al., 1983; Billger et al., 1988), and execute axonal degeneration (Yang et al., 2013). Increased calpain activation is observed in both MS plaques and normal-appearing-white matter (NAWM; Shields et al., 1999), and inhibitors of calpains reduce axonal and neuronal damage in the context of EAE (Hassen et al., 2008; Smith et al., 2011). Demyelinated axons may, therefore, be particularly vulnerable to calcium overload and subsequent degeneration, especially when ATP production is limited and the NCX reverses (Figure 3).

### Entryways for Extracellular Ca^2+^

Identifying and inhibiting sources of axoplasmic calcium accumulation may be a protective strategy in demyelinating disease. Extracellular Ca^2+^ can reach concentrations as high as 1.5 mM which is approximately 15,000× greater than the resting neuronal Ca^2+^ concentration of ∼100 nM (Gleichmann and Mattson, 2011). This produces a strong gradient favoring movement of Ca^2+^ into the neuron. External influx of Ca^2+^ is typically regulated through voltage-gated calcium channels (VGCCs), glutamate receptors and non-selective cation channels (Stirling and Stys, 2010; Gleichmann and Mattson, 2011; Friese et al., 2014). During demyelination in both EAE and MS, in addition to L-type calcium channels which are typically found along axons, N-type calcium channels are upregulated and are associated with axonal swelling (Kornek et al., 2001; Gadjanski et al., 2009). Blockade of N or L-type calcium channels ameliorates EAE (Brand-Schieber and Werner, 2004; Gadjanski et al., 2009; Tokuhara et al., 2010; Ingwersen et al., 2018) and protects axons (Gadjanski et al., 2009; Ingwersen et al., 2018). There is also evidence that glutamate receptors may permit Ca^2+^ entry into demyelinated neurons. In MS, there is an elevation of glutamate in the brain (Srinivasan et al., 2006), potentially caused by release from dying neurons and glia, inflammatory cells (Pampliega et al., 2011) or via impaired uptake (Geurts et al., 2003; Cambron et al., 2012). When activated at prodigious levels glutamate receptors can result in Ca^2+^ entry and excitotoxicity. Accordingly, axonal degeneration is reduced in adoptive-transfer EAE when the AMPAR antagonist NBQX is administered (Pitt et al., 2000), and protects neurons in MBP-induced EAE in rats (Smith et al., 2000). Likewise, NMDAR antagonists like memantine, MK-801 and fullerene ABS-75 are axon-protective in EAE (Basso et al., 2008; Farjam et al., 2014; Levite, 2017). This demonstrates that glutamate or VGCC blockade can be effective at alleviating axon loss following inflammatory demyelination. A caveat is that glutamate and VGCCs have potent effects on inflammatory activation in EAE (Smith et al., 2000; Basso et al., 2008; Fallarino et al., 2010; Tokuhara et al., 2010; Sulkowski et al., 2013; Fazio et al., 2014; Ingwersen et al., 2018) making it difficult to ascertain how much of the neuroprotection is derived by a direct alleviation of calcium overload in the axon relative to immunomodulation. Administration of VGCC inhibitors during EAE does not diminish Ca^2+^ levels in the axon when examined using a genetic indicator of intracellular calcium, arguing against a direct axonal-protective effect (Witte et al., 2019). Likewise, application of glutamate agonists are not sufficient to drive axonal Ca^2+^ in the spinal cord axons (Witte et al., 2019). This argues that immune modulation may be responsible for VGCC and glutamate-mediated axonal protection rather than via direct modulation of Ca^2+^ levels in the axon. Interestingly, axons in EAE are permeable to extracellular molecules of up to 10 kDa in size indicative of small, non-specific ruptures (<10 nm) in the axoplasmic membrane. The cause of these “nano-ruptures” in the membrane is unclear but are associated with inflammation, and inflammatory cells are known to secrete membrane rupturing proteins like perforins and complement (Zhao et al., 2018). This presents a novel axis by which Ca^2+^ can enter the axon – through direct damage to the membrane. Interestingly, myelinated axons had reduced permeability of 10 kDa molecules suggestive of fewer nano-ruptures (Witte et al., 2019). A shielding effect of myelin on the axon from immune mediators may be another mechanism by which oligodendrocytes protect axons. Whether nano-ruptures occur or constitute a major source of intra-axonal calcium in the absence of auto-immunity would be interesting to test in chemical or genetic models of demyelination.

### Mitochondria Contribute to Axonal Calcium Overload

There are two main organelles which store calcium; the endoplasmic reticulum (ER) and mitochondria. Release of these internal sources of Ca^2+^ could contribute to calcium overloading in demyelinating disease. During excitotoxic injury (Ouardouz et al., 2009), axotomy (Stirling et al., 2014; Villegas et al., 2014), or oxygen-glucose deprivation (Ouardouz et al., 2003), ER Ca^2+^ release contributes to axoplasmic calcium overloading and subsequent degeneration. However, the depletion of endoplasmic stores with caffeine does not induce axoplasmic Ca^2+^ rises in axons in EAE (Witte et al., 2019), and to our knowledge there is no direct evidence demonstrating axoplasmic Ca^2+^ contributes to axonal degeneration during demyelination. However, mitochondria likely do contribute to axoplasmic Ca^2+^ dynamics. The mitochondria calcium uniporter (MCU) is the primary Ca^2+^ transporter involved with buffering calcium in mitochondria. The MCU increases uptake during times of high cytoplasmic Ca^2+^ and low ATP/ADP ratio (Igbavboa and Pfeiffer, 1988; Litsky and Pfeiffer, 1997; Baughman et al., 2011; De Stefani et al., 2011; Gleichmann and Mattson, 2011). Ca^2+^ uptake into the mitochondria increases ATP synthesis by activating dehydrogenases that make reducing equivalents, which drive complex I activity (Denton et al., 1972; Nichols et al., 2017). Therefore, Ca^2+^ influx into mitochondria may serve two important roles: to reduce cytoplasmic calcium accumulation and to increase ATP production. Deletion of the MCU from neurons following induction of MOG_35__–__55_ EAE, exacerbates EAE severity, reduces ATP content in the spinal cord and increases axonal damage (Holman et al., 2020). More T-cells and myeloid cells are found in mice with neuron-specific deletion of MCU (Holman et al., 2020), indicating that heightened inflammatory activity can be secondary to potentiated neuronal damage. If calcium buffering in mitochondria is critical for axonal health, increased efflux of Ca^2+^ from the mitochondria should leave axons vulnerable to degeneration in demyelinating disease. The mitochondrial permeability transition pore (MPTP) can drive the efflux of calcium from the mitochondria, and is regulated by cyclophilin D (Connern and Halestrap, 1996; Nicolli et al., 1996). The absence of cyclophilin D makes the MPTP threshold for opening higher and would be predicted to make the axon more resistant to damage during demyelination. Germline cyclophilin D knockout mice had less damage and reduced EAE severity (Forte et al., 2007). Inflammatory infiltration of T-cells or monocytes is not inhibited, suggestive of a direct effect on neuronal health (Forte et al., 2007). A pharmacological inhibitor of the MPTP also reduced EAE severity and decreased axonal damage (Warne et al., 2016). Taken together, mitochondrial calcium uptake has an important role in reducing neuronal damage following demyelination, likely by ameliorating calcium rise within the axoplasmic compartment and by increasing ATP production.

### Sodium Channel Activation Is Associated With Axonal Degeneration Following Demyelination

The increased expression of sodium channels along demyelinated axons raises their metabolic demand and places increased energetic stress on the axon. In both EAE and in MS lesions, Na_v_1.6 channels colocalize with the NCX (Craner et al., 2003, 2004), which under conditions of energetic stress imports Ca^2+^ in order to extrude Na^+^ (Figure 3; Stys et al., 1991, 1992). This posits a mechanism by which Na^+^ accumulation during an energetic rundown can directly contribute to Ca^2+^ buildup. Axons with N_av_1.6 and NCX colocalization are much more prone to βAPP expression in MS, a marker of transport deficit and axonal damage (Craner et al., 2003, 2004). Broad pharmacological inhibitors of sodium channels during EAE reduce axonal damage and motor impairment (Lo et al., 2003; Bechtold et al., 2004, 2006; Black and Waxman, 2008; Morsali et al., 2013; Al-Izki et al., 2014). However, from these studies it was unclear if sodium blockade diminished axonal damage by acting directly on the axon or via its known role in diminishing microglia/macrophage infiltration and activation within the CNS (Craner et al., 2005; Black et al., 2009; Morsali et al., 2013). Cell-specific targeting of voltage-gated sodium channels in neurons demonstrates that sodium channels can act directly on neurons to drive their damage in EAE. Using adeno-associated virus (AAV) deletion of Na_v_1.6 in RGCs following EAE, greater preservation of axons in the optic nerve is observed as well as reduced neuronal loss (Alrashdi et al., 2019). Unfortunately, clinical trials of sodium channel blockers have been less promising with early clinical trials in those with SPMS using lamotrigine having found diminished brain volume relative to placebo over the first year before stabilizing (Kapoor et al., 2010; Hayton et al., 2012). Brain volume measures can be confounded by inflammatory infiltrate and edema, which is likely reduced by sodium channel blockade and could have contributed to this decline in brain volume before stabilization (Franklin et al., 2012). A recent clinical trial using optical coherence tomography (OCT) to measure neurodegeneration within the retinal nerve fiber layer (RNFL) in those with optic neuritis found that treatment with the sodium channel blocker phenytoin resulted in a 30% reduction in the thinning of the RNFL and increased macular volume after six months (Raftopoulos et al., 2016). This study highlights that voltage-gated sodium blockade may provide some level of neuroprotection, at least during RRMS. However, it is still unclear the precise mechanism(s) by which sodium blockade confers axon protection and if it contributes to calcium overloading following demyelination.

### Axon Transport Deficits Following Demyelination Impair Energy Production in the Axon Leaving It Vulnerable to Degeneration

The axon represents a logistical challenge unique amongst any cell-type; mRNA, proteins and organelles produced in the soma must be trafficked for vast distances. For example, corticospinal neurons can have an axon over 1 m long, exceeding the size of its soma by 50,000 times. Mitochondria require nuclear genes in addition to their own genome for proper function, and mitochondrial biogenesis mostly occurs in the soma (Calvo et al., 2016). Mitochondria are then trafficked to meet local energy requirements along the axon (Misgeld and Schwarz, 2017; Campbell et al., 2019). This logistical bottleneck along with proximity to inflammation in the axon, can make mitochondria highly sensitive to damage and dysfunction in demyelinating lesions, which presents an axis of vulnerability especially in long axons.

In EAE, both anterograde and retrograde deficits in mitochondrial transport are detected, with anterograde transport more adversely affected (Sorbara et al., 2014). Transport deficits precede axonal blebbing and outright degeneration (Sorbara et al., 2014). Inflammatory mediators like reactive oxygen species (ROS) and reactive nitrogen species (RNS) directly impair transport of mitochondria following EAE, evident by the restoration of transport when ROS/RNS scavengers are administered (Sorbara et al., 2014). Another potential inhibitor of axonal transport in demyelinated axons includes excitotoxicity from glutamate (or TNFα exposure), which causes a calcium-dependent relocalization of histone deacetylase 1 (HDAC1) from the nucleus to the axon (Kim et al., 2010). Axonal HDAC1 interacts with the kinesin family of motor proteins where it hinders their interaction with cargo such as mitochondria to diminish their transport (Kim et al., 2010). Downregulation of HDAC1 or preventing its translocation from the nucleus reduces axonal damage following glutamate exposure (Kim et al., 2010).

If reduced transport of mitochondria to the axon is associated with axonal damage can increasing transport be protective? Overexpression of the protein Miro1, which tethers mitochondria to their motor adaptor complex, or mitochondrial biogenesis peroxisome proliferator activated receptor gamma coactivator 1-alpha (PGC1-α) are effective at increasing anterograde transport and reducing axonal damage following demyelination of cerebellar slice cultures (Licht-Mayer et al., 2020). Considering that kinesins require ATP hydrolysis to move cargoes such as mitochondria through the axons, a positive feedback loop may occur where the axon does not have the energy to effectively translocate the mitochondria to meet localized energy production, which in turn further impairs energy production at distal sites. This may explain why mitochondria content falls in distal component of the axon in chronic MS and EAE (Dutta et al., 2006; Sorbara et al., 2014). A failure of energy production at the synapses, which require considerable energy for neurotransmission (Lennie, 2003), may contribute to their loss/dysfunction in MS and demyelinating models (Jurgens et al., 2016; Werneburg et al., 2020).

### Mitochondrial Damage and Dysfunction Following Demyelination

Microglia/macrophages closely appose axons during inflammatory demyelination (Nikic et al., 2011), and produce ROS and RNS which can impair and damage mitochondria. The best studied reactive species in the context of demyelination is nitric oxide (NO). NO is not inherently cytotoxic and has many important physiological roles including mediating vasodilation. However, when combined with superoxide it forms the toxic peroxynitrate which oxidizes tyrosine residues and damages proteins (Pacher et al., 2007). Importantly, NO also binds the ferrous heme of cytochrome c oxidase (mitochondrial complex IV) drastically reducing mitochondrial respiration (Brown and Cooper, 1994; Cleeter et al., 1994). Inducible nitric oxide synthase (iNOS) expression and nitrotyrosine residues are found in active MS lesions and the active edge of chronic MS lesions (Bo et al., 1994; Cross et al., 1996; Oleszak et al., 1998; Lu et al., 2000; Liu et al., 2001; Marik et al., 2007). Early active lesions in MS have selectively reduced activity of complex IV (Mahad et al., 2008), whose expression and functionality can be directly targeted by NO (Brown and Cooper, 1994; Cleeter et al., 1994; Wei et al., 2002). NO exposure blocks conduction in demyelinated axons (Redford et al., 1997) and results in axonal degeneration during high frequency stimulation (Smith et al., 2001; Kapoor et al., 2003). The selective vulnerability of axons during high frequency stimulation suggests that NO disrupts energy production, which leaves axons under intense energetic burden to remove excess sodium, which may culminate in the reversal of the NCX. In agreement with this hypothesis, sodium channel blockers and NCX inhibitors were shown to have a protective effect and ameliorate axonal degeneration following NO administration (Kapoor et al., 2003). Live-imaging studies during EAE provide further evidence that inflammation triggers mitochondrial dysfunction during demyelination, in part through a NO-mediated mechanism. Fluorescently labeled mitochondria along with potentiometric dyes were used to determine that with the onset of inflammation there is a collapse of axonal mitochondrial membrane potential, indicative of mitochondrial dysfunction (Sadeghian et al., 2016). Both hydrogen peroxide and NO treatment to spinal axons induced swelling of mitochondria within axons (Nikic et al., 2011), and treatment with ROS/RNS species scavengers attenuates mitochondrial swelling and axonal degeneration during EAE (Nikic et al., 2011). However, it was not determined if NO specifically is causative in impairing mitochondrial respiration in these studies of EAE. It also remains unclear if mitochondrial dysfunction induced by ROS/RNS plays a major role during the chronic phases of MS when acute demyelinating lesions become rare or in demyelinating diseases that do not have considerable inflammatory infiltrate, such as inherited leukodystrophies.

Mitochondria damage continues to accrue during chronic demyelination. Mitochondrial DNA (mtDNA) lacks protective histones and some DNA repair enzymes making it vulnerable to damage (Yakes and Van Houten, 1997; Calvo et al., 2016; van den Berg et al., 2017) and oxidative damage to mtDNA is detected in MS (Haider et al., 2011). With disease chronicity there is an accumulation of cortical neurons with deficient Complex IV respiration, which is encoded in part by mtDNA (Dutta et al., 2006; Zambonin et al., 2010; Campbell et al., 2011), whereas nuclear-encoded Complex II respiration is often intact, a common characteristic of mitochondrial diseases (DiMauro and Schon, 2003). Dissecting these respiratory deficient neurons specifically, some studies found they had a high rate of mtDNA deletions, which may be further amplified over time via clonal expansion (Campbell et al., 2012). Mitochondrial injury and respiratory chain dysfunction lead to the liberation of more electrons which can then subsequently react with oxygen and induce more ROS-mediated damage. This constitutes a positive feedback loop that can increasingly imperil energy production in the neuron. Damaged mitochondria are removed by mitophagy, a process that requires fusion of the mitochondrial membrane with the lysosome in the soma or potentially to some extent within the axon (Misgeld and Schwarz, 2017). Expression of synaptophilin, necessary to anchor mitochondria to demyelinated internodes (Ohno et al., 2014), also impairs their transport and renders mitochondria unable to be effectively degraded following chronic stress (Lin et al., 2017). In this sense, increased energy demand during chronic demyelination coincides with accumulating damage and decreased elimination of dysfunctional mitochondria. Unfortunately, the long-term oxidative damage to mitochondria and failure of the respiratory chain observed in progressive MS is not typically detected in rodent models (Schuh et al., 2014), making it challenging to use these experimental models to assess to what extent mitochondrial dysfunction drives degeneration.

### Are Intra-Axonal Signaling Cascades Important for Wallerian Degeneration Involved in Axonal Degeneration in Demyelinating Disease?

The term Wallerian degeneration is used to describe the process by which the distal axon degenerates following injury along with the subsequent activation of glia (Waller, 1851; Gaudet et al., 2011; Wang et al., 2012). The discovery of a spontaneous mutant, the slow-Wallerian degeneration mouse (*Wld*^*s*^) that greatly delays axonal degeneration following transection (Lunn et al., 1989), has permitted a detailed understanding of the molecular mechanisms underlying Wallerian degeneration. A comprehensive description of theses mechanisms is beyond the scope of this review and has been recently covered in a number of excellent reviews (Freeman, 2014; Gerdts et al., 2016; Coleman and Hoke, 2020). However, many of the proteins identified contribute to axon and neuron loss in neurodegenerative diseases like ALS and AD, as well as potentially in demyelinating diseases (Coleman and Hoke, 2020). Indeed, there is considerable evidence of Wallerian-like degeneration following demyelination in MS. Diffuse axonal damage and degeneration is observed distal to lesions throughout the NAWM early during the disease (Kornek et al., 2000; Filippi et al., 2003) consistent with transected distal axons in acute lesions undergoing Wallerian degeneration. Additionally, Neuropeptide Y1-receptor, a marker for transected axons undergoing Wallerian degeneration (Pesini et al., 1999), is detected in MS lesions and NAWM (Dziedzic et al., 2010; Singh et al., 2017). To determine if Wallerian degeneration has a functional role in disease presentation or axonal degeneration in EAE, Chitnis et al. (2007) induced EAE in *Wld*^*s*^ mice. *Wld*^*s*^ mice initially have reduced axonal degeneration and disease severity, but disease severity worsens overtime to be no different than controls (Chitnis et al., 2007). While the authors did not examine axonal degeneration at that later time point, a recent study examining the knockout of *Sarm1*, a downstream effector necessary for Wallerian degeneration (Osterloh et al., 2012), in EAE found diminished axon damage early but no long-term benefit on axonal health (Viar et al., 2020). Taken together, Wallerian degeneration contributes to axonal loss in MS and EAE, but *Wld*^*s*^ and related proteins do not seem to confer long-term axonal protection against immune-mediated demyelination.

The failure to deliver a critical survival factor following axotomy to the axon from the soma was proposed decades ago by Lubinska to be the trigger for Wallerian degeneration (Lubinska, 1982). In 2010, such a protein was identified (Gilley and Coleman, 2010). Nicotinamide mononucleotide adenylyltransferase 2 (NMNAT2), is a highly labile protein necessary for the maintenance of the axon that is anterogradely transported and rapidly degraded following axotomy (Gilley and Coleman, 2010). NMNAT2 regulates NAD biosynthesis, which is critical for redox reactions necessary to maintain mitochondrial respiration and ATP production. This suggests that proteins critical to inhibiting Wallerian degeneration intersect with, and are necessary for, energy homeostasis. Given that axonal energy deficiency is linked to axon loss in demyelinating disease (Trapp and Stys, 2009), it is plausible that bolstering NMNAT or NAD levels may be an effective approach to enhance axon protection following demyelination. NAD levels decline during EAE and supplying NAD or its precursors diminishes demyelination and axonal damage (Kaneko et al., 2006; Tullius et al., 2014). However, NAD is also a major immune-regulatory molecule and can modulate T-cell infiltration and differentiation following EAE (Kaneko et al., 2006; Tullius et al., 2014). Future work should determine if NAD acts directly on axons following demyelination, or if axonal protective effects are primarily through immune-modulation. Either way, the finding that NAD protects against axonal degeneration in EAE is intriguing and potentially offers a new axis for treatments that protect against axonal degeneration.

Studies of Wallerian degeneration have also revealed active ‘death signaling’ within the axon is necessary for axonal degeneration (Wang et al., 2012; Llobet Rosell and Neukomm, 2019). Inhibition of mitogen-associated kinases (MAPKs), especially dual leucine zipper kinase (DLK), protect axons following axotomy, and likely constitutes such a death signal (Miller et al., 2009). Interestingly, MAPK signaling intersects with NAD biosynthesis as activated MAPK signaling promotes NMNAT2 turnover (Walker et al., 2017). Likewise, the downregulation of DLK along with the related leucine zipper kinase (LZK) preserves NMNAT2 levels and neurites *in vitro* (Summers et al., 2018). At this time, there is no direct evidence that DLK is regulated or MAPKs are phosphorylated following demyelination, however there is considerable reason to believe these pathways may be involved. Axonal transport is rapidly affected by inflammatory demyelination (Sorbara et al., 2014) and DLK inhibition protects against insults to fast axonal transport imparted by vincristine (Miller et al., 2009; Summers et al., 2018). Additionally, when mitochondria respiration is inhibited (such as by sub-lethal doses of the ATPase inhibitor oligomycin) NMNAT2 levels fall and activation of DLK signaling drives axonal degeneration (Summers et al., 2020). If MAPK signaling is activated following demyelination, it may serve as druggable target with overlap to other neurodegenerative disorders like ALS and AD where targeting of DLK reduces neurodegeneration in rodent models (Le Pichon et al., 2017).

## Conclusion and Future Perspectives

The interaction between the oligodendrocyte and axon is critical for axonal structure and function, which is necessary for the rapid and timely propagation of action potentials throughout the CNS. The loss of myelin and oligodendrocytes fundamentally alters the neuron. Neurons restructure their excitatory domains, increase their mitochondrial content, and undergo significant transcriptional changes in response to demyelination. While these adaptations may allow some degree of functional restoration and conductance, it ultimately leaves the axon vulnerable to damage. Demyelinated axons that lack oligodendrocyte support are susceptible to energetic failure and the accumulation of intracellular calcium, which drives subsequent degeneration.

While there has been much progress in recent years in understanding how oligodendrocytes support axons in health and disease, a number of critical questions remain. Given numerous clinical trials are beginning with the aim of improving remyelination, it will be crucial to determine whether remyelinating oligodendrocytes provide long-term support of axonal health and how they differ from those produced during development. Are core functions of oligodendrocytes such as potassium buffering and metabolic support fully restored following remyelination? Already, single-cell RNA sequencing are providing clues and finding distinctions between oligodendrocytes formed in development and those during remyelination (Falcao et al., 2018; Jakel et al., 2019). Likewise new tools available to neuroscientists will be helpful in unraveling how demyelinated neurons degenerate. While it is clear that calcium overload drives axonal degeneration during acute inflammatory demyelination (Witte et al., 2019), determining the extent to which energetic rundown and calcium accumulation drives degeneration in chronically demyelinated axons is of pressing concern. Advances in two-photon live-imaging along with genetically encoded sensors for ATP, calcium and other molecules in demyelinating axons will be crucial tools for answering this question and determining the mechanisms by which calcium accumulates and mitochondria are damaged (Trevisiol et al., 2017; Looser et al., 2018). Likewise, genetic models of demyelination, which offer cell-specificity and the potential to impair subsequent remyelination (Duncan G. J. et al., 2017), will be important for delineating how neurons respond to demyelination, which changes are protective, and for identifying neuroprotective targets. The breakdown of oligodendrocyte-axon interactions is seen in a variety of neurological disorders beyond the classical demyelinating diseases; a further understanding of the molecular mechanisms by which demyelinated axons degenerate will likely offer novel therapeutic insights to alleviate decline in these pathologies.

## Author Contributions

GD and BE contributed to the conception and design of the review. GD prepared the figures. All authors contributed to the writing, editing, and approval of the submitted manuscript.

## Conflict of Interest

BE is a co-founder of Autobahn Therapeutics. The remaining authors declare that the research was conducted in the absence of any commercial or financial relationships that could be construed as a potential conflict of interest.
